# PGPR-Mediated Salt Tolerance in Maize by Modulating Plant Physiology, Antioxidant Defense, Compatible Solutes Accumulation and Bio-Surfactant Producing Genes

**DOI:** 10.3390/plants11030345

**Published:** 2022-01-27

**Authors:** Baber Ali, Xiukang Wang, Muhammad Hamzah Saleem, Sumaira, Aqsa Hafeez, Muhammad Siddique Afridi, Shahid Khan, Zaib-Un-Nisa, Izhar Ullah, Antônio Teixeira do Amaral Júnior, Aishah Alatawi, Shafaqat Ali

**Affiliations:** 1 Department of Plant Sciences, Quaid-i-Azam University, Islamabad 45320, Pakistan; baberali@bs.qau.edu.pk (B.A.); khanzadi.tipu@gmail.com (A.H.); izharullah352@gmail.com (I.U.); 2 College of Life Sciences, Yan’an University, Yan’an 716000, China; 3 College of Plant Science and Technology, Huazhong Agricultural University, Wuhan 430070, China; saleemhamza312@webmail.hzau.edu.cn; 4 Department of Biotechnology, Quaid-i-Azam University, Islamabad 45320, Pakistan; sumaira.khan1890@gmail.com; 5 Department of Plant Pathology, Federal University of Lavras (UFLA), Lavras 37200-900, MG, Brazil; msiddiqueafridi@gmail.com; 6 Department of Agriculture, University of Swabi, Ambar, Swabi 94640, Pakistan; shahidkhan@aup.edu.pk; 7 Laboratório de Melhoramento Genético Vegetal, Centro de Ciências e Tecnologias Agropecuárias, Universidade Estadual do Norte Fluminense Darcy Ribeiro (UENF), Campos dos Goytacazes 28013-602, RJ, Brazil; amaraljr@uenf.br; 8 Cotton Research Institute, Multan 60000, Pakistan; zaibsci.agri@gmail.com; 9 Biology Department, Faculty of Science, University of Tabuk, Tabuk 71421, Saudi Arabia; Amm.alatawi@ut.edu.sa; 10 Department of Environmental Sciences, Government College University, Faisalabad 38000, Pakistan; 11 Department of Biological Sciences and Technology, China Medical University, Taichung 40402, Taiwan

**Keywords:** abiotic stresses, agriculture, halo-tolerant bacteria, plant-microbe interactions, salinity stress

## Abstract

Salinity stress is a barrier to crop production, quality yield, and sustainable agriculture. The current study investigated the plant growth promotion, biochemical and molecular characterization of bacterial strain *Enterobacter cloacae* PM23 under salinity stress (i.e., 0, 300, 600, and 900 mM). *E. cloacae* PM23 showed tolerance of up to 3 M NaCl when subjected to salinity stress. Antibiotic-resistant Iturin C *(ItuC*) and bio-surfactant-producing genes (*sfp and srfAA*) were amplified in *E. cloacae* PM23, indicating its multi-stress resistance potential under biotic and abiotic stresses. Moreover, the upregulation of stress-related genes (APX and SOD) helped to mitigate salinity stress and improved plant growth. Inoculation of *E. cloacae* PM23 enhanced plant growth, biomass, and photosynthetic pigments under salinity stress. Bacterial strain *E. cloacae* PM23 showed distinctive salinity tolerance and plant growth-promoting traits such as indole-3-acetic acid (IAA), siderophore, ACC deaminase, and exopolysaccharides production under salinity stress. To alleviate salinity stress, *E. cloacae* PM23 inoculation enhanced radical scavenging capacity, relative water content, soluble sugars, proteins, total phenolic, and flavonoid content in maize compared to uninoculated (control) plants. Moreover, elevated levels of antioxidant enzymes and osmoprotectants (Free amino acids, glycine betaine, and proline) were noticed in *E. cloacae* PM23 inoculated plants compared to control plants. The inoculation of *E. cloacae* PM23 significantly reduced oxidative stress markers under salinity stress. These findings suggest that multi-stress tolerant *E. cloacae* PM23 could enhance plant growth by mitigating salt stress and provide a baseline and ecofriendly approach to address salinity stress for sustainable agriculture.

## 1. Introduction

Abiotic and biotic stresses affect plant growth, yield, and productivity [1]. Salinity stress is the most detrimental abiotic stress that severely affects agricultural productivity and global food security [2]. Salinity affects approximately 62 million hectares (20%) of the agricultural area [3]. It is predicted that by 2050, more than half of irrigated land will be saline [4].

Salinity affects seed germination, plant growth, and development, resulting in major agricultural yield losses worldwide [5]. All growth phases, including germination, seedling, vegetative, and mature stages, have been reported to undergo morphological changes under saline stress [6]. Salt stress causes various biochemical changes, including antioxidant enzyme activation, modulation of phytohormones, changes in ion uptake, generation of reactive oxygen species (ROS), and disruption of photosynthetic pathways [3]. Furthermore, microbial activities, e.g., soil respiration and enzymatic activities, were inhibited by salinity stress. As a result, soil salinization is universally recognized as a severe hazard to agriculture [7].

To mitigate salinity-induced challenges in the future, an urgent need for alternative eco-friendly technologies is necessary, such as the use of plant growth-promoting microorganisms (PGPM). A rhizospheric microbiome is a diverse group of soil microbes that form a symbiotic association with plants and could play an important role in salinity stress [8]. Plant growth-promoting bacteria (PGPB) elicit salinity tolerance and positively affect plant life cycles, promoting plant growth by direct and indirect mechanisms [9]. Plant growth-promoting bacteria mostly inhabit the root zone in saline soil, establishing specific symbiotic relationships with plants. Plant growth-promoting bacteria promote plant growth by employing direct mechanisms such as nutrient acquisition, siderophore sequestration, nitrogen fixation, synthesis of exopolysaccharides and phytohormones, and potassium and phosphate solubilization under salinity stress [10]. The plant growth improvement by Plant growth-promoting bacteria under stress conditions involves other mechanisms such as upregulation of plant antioxidant defense enzymes to protect the plant against oxidative damage [11].

Maize (*Zea mays* L.) is moderately salt-sensitive and one of the most important cereal crops [12]. After wheat and rice, maize (*Zea mays* L.) is Pakistan’s third most important cereal [13]. The application of PGPB in maize plants leads to a significant increase in nutrient acquisition and prevents extra salt ions transportation into the plant tissue, improving salt tolerance. Furthermore, treatment with two plant growth-promoting bacteria that produce ACC deaminase (*Kocuria rhizophila* 14ASP) alleviated the adverse effects of salt stress in wheat plants [14,15]. Moreover, salt-tolerant Plant growth-promoting bacteria increases proline content, regulating phytohormone levels, nutrient acquisition, redox potential, ion homeostasis, photosynthetic capacity, and stress-responsive gene expression, which are positively correlated to salt tolerance [16,17].

Under salinity stress, salt-tolerant *Pseudomonas fluorescens* promotes plant growth in maize [12]. Similarly, *Enterobacter* sp. P23, which produce exopolysaccharide, helps rice seedlings to cope with salinity stress [18]. *Bacillus subtilis* mitigates salinity stress in white clover [19]. In wheat, inoculation with *Bacillus aquimaris* tolerates salt stress and promotes plant growth [20]. Egamberdieva et al. (2017) have reported the role of *Pseudomonas extremorientalis* TSAU20 in salinity-stressed tomato plants [21]. Inoculation of *Azotobacter chroococcum* has been reported to mitigate salt stress in plants [22]. Exopolysaccharides produced by Plant growth-promoting bacteria can stimulate plant growth in salt-stressed conditions [23]. Salt-tolerant *Bacillus* strains were isolated, characterized, and can stimulate the growth and development of plant species [24]. Egamberdiyeva [25] found that Plant growth-promoting bacteria *Bacillus polymyxa* BcP26 can mitigate salt stress and nutrient acquisition of maize in calcisol soil, so effectively help in alleviating salt stress.

In our research, rhizospheric bacteria *Enterobacter cloacae* PM23 was investigated regarding salinity tolerance, plant growth-promoting (PGP) activities, and its ability to alleviate salt stress in maize plants. The current research aims to investigate the biochemical and molecular responses of *E. cloacae* PM23 tolerance and its effects on maize growth under various salinity stress. The *in-vitro* and pot experiment revealed that *E. cloacae* PM23 as a salt-tolerant plant growth-promoting rhizobacteria could potentially improve plant growth and alleviate salt stress by regulating molecular and biochemical mechanisms in salt stress lands.

## 2. Results

### 2.1. Growth Curve Analysis of Enterobacter cloacae PM23

**Figure 1:**
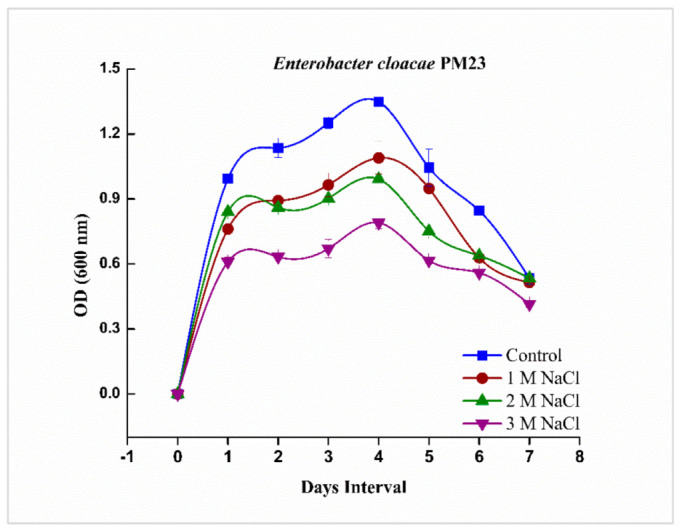
Growth curve analysis of *E. cloacae* PM23 under salinity stress (0, 1, 2 and 3 M NaCl).

It was investigated that *E. cloacae* PM23 can survive under salinity stress up to 3 M NaCl concentration in Luria-Bertani (LB) medium. Growth curve analysis revealed a log phase at the 4th day of incubation (Figure 1).

### 2.2. Salinity Tolerance Traits of E. cloacae PM23 under Salinity Stress

**Figure 2:**
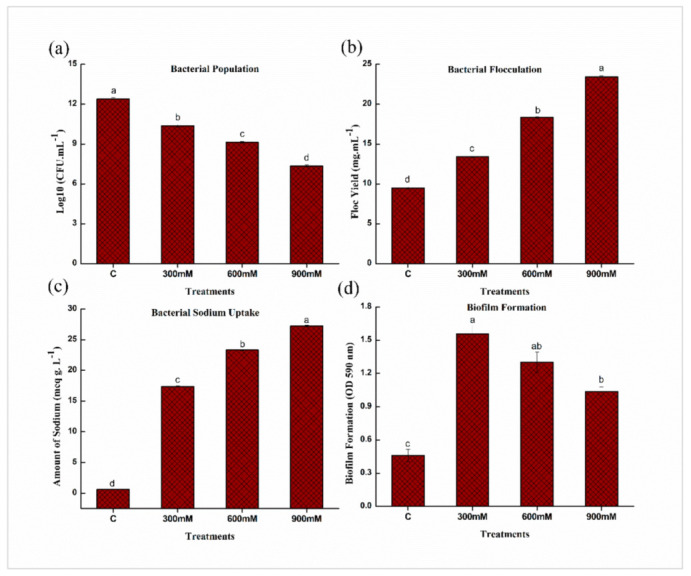
Effects of NaCl on salinity tolerance traits of *E. cloacae* PM23 (**a**) Bacterial population (**b**) Flocculation yield (**c**) Bacterial sodium uptake (**d**) Biofilm formation.

As salinity stress was increased from 300 to 900 mM NaCl, the population of bacterial cells and biofilm formation significantly decreased from 10.36 to 7.35 CFU/mL and 1.55 to 1.03 compared to control (Figure 2a,d). There was a substantial drop in population and biofilm formation at 900 mM NaCl, with respect to control. While bacterial floc yield and sodium uptake gradually increased from 13.42 to 23.43 mg/mL and 17.38 to 27.24 meq/mL, respectively, as salinity stress was increased from 300 to 900 mM NaCl with respect to control (Figure 2).

### 2.3. Quantitative Estimation of Plant Growth Promoting (PGP) Traits of E. cloacae PM23

**Figure 3:**
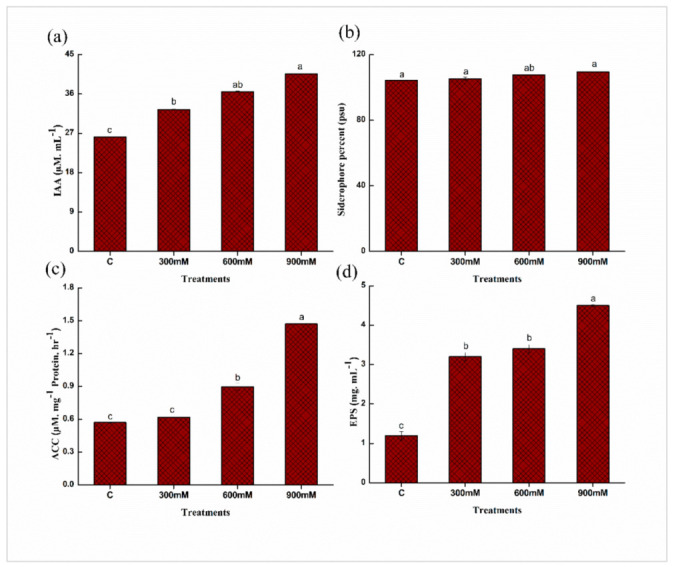
Quantitative estimation of PGP traits of *E. cloacae* PM23: (**a**) Indole-3-acetic acid (IAA) (**b**) Siderophore (**c**) ACC deaminase (ACCD) (**d**) Exopolysaccharides (EPS).

To investigate the potential of *E. cloacae* PM23 under salinity stress (0, 300, 600, and 900 mm NaCl), plant growth-promoting attributes were assessed. The results revealed that indole-3-acetic acid (IAA) production increased substantially with increasing salinity stress, and the highest IAA production (35%) was observed at 900 mM NaCl compared to the control. Similarly, at 900 mM NaCl, siderophore production (5%), ACC Deaminase (ACCD) activity (61%), and exopolysaccharides (EPS) (73%) production increased as compared to the control (Figure 3).

### 2.4. Physio-Chemical Properties of Soil

Table 1 shows the physicochemical properties of soil. The soil texture of both soils (Pre-sowing and Post-harvesting) were loamy, slightly alkaline, and had an electrical conductivity of 1.53 and 4.49 dS/m, respectively. Organic matter present in pre-sowing is higher than post-harvested soil, while saturation was higher in pre-sowing soil than post-harvested soil. However, available phosphorus and potassium were higher in content in pre-sowing soil than post-harvested soil.

### 2.5. Effects of E. cloacae PM23 on Biomass and Growth of Zea mays L.

After 21 d of sowing, maize plants were harvested to observe the agro-morphological attributes of all treatments. All parameters including shoot, root length, the height of the plant, fresh, dry weight, and total leaf surface area were significantly reduced under salinity stress compared to control (Table 2). The results revealed the promising effects of *E. cloacae* PM23 inoculation on all parameters of maize plants under normal as well as salinity stress (300, 600, and 900 mM NaCl). The shoot (21–36%), root length (28–45%), and height (23–39%) of maize plants were significantly increased with the treatment of *E. cloacae* PM23 under salinity stress as compared to respective controls (Table 2). A similar trend in biomass (Fresh weight: 40–51%; Dry weight: 46–58%) were noted but there was no significant differences induced by *E. cloacae* PM23 under the salinity stress. While total leaf surface area (21–29%) of maize plants was significantly increased with inoculation of *E. cloacae* PM23 under salinity stress as compared to their un-inoculated controls (Table 2).

### 2.6. Effects of E. cloacae PM23 on Pigmented Content, Carotenoids, and Relative Water Content of Maize Plants

Pigmented content (chl a, b, and total chl), carotenoids, and relative water content of maize plants showed a significant reduction under salinity stress (Table 3). While *E. cloacae* PM23-treated plants significantly enhanced the pigmented content (chl a: 31–37%; chl b: 11–31%; total chl: 26–31%), carotenoids (14–30%) and relative water content (13–23%) (Table 3). By inoculating *E. cloacae* PM23, radical scavenging activity of leaves (DPPH) significantly increased (13–26%) under salinity stress (300, 600, and 900 mM NaCl) (Table 4). All results were compared with their respective controls (Table 4).

### 2.7. Effects of E. cloacae PM23 on Level of Antioxidant Enzymes

**Figure 4:**
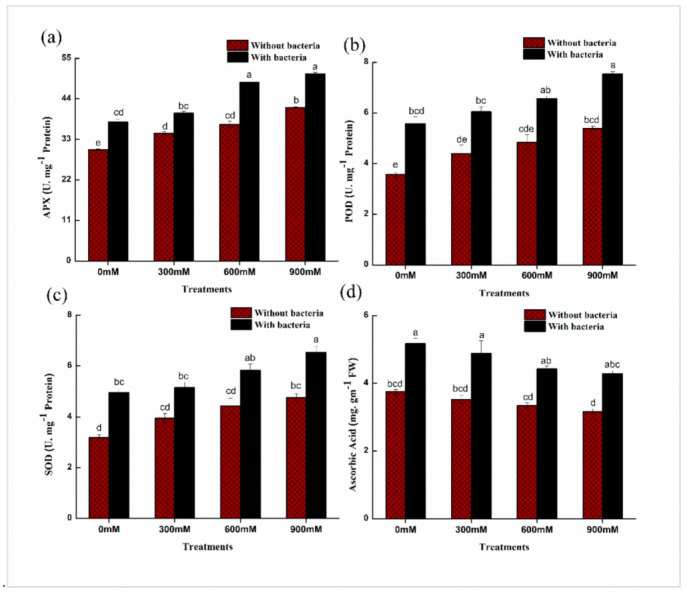
Effects of *E. cloacae* PM23 on levels of enzymatic and non-enzymatic antioxidants: (**a**) Ascorbate peroxidase (APX) (**b**) Peroxidase (POD) (**c**) Superoxide dismutase (SOD) (**d**) Ascorbic Acid.

The oxidative stress ameliorating response was determined by estimating the activity of various enzymes such as ascorbate peroxidase (APX), peroxidases (POD), superoxide dismutase (SOD), and ascorbic acid (AA) in plants. The activity of these enzymes (APX, POD, and SOD) increased non-significantly while ascorbic acid decreased under salinity (0, 300, 600, and 900 mM NaCl) (Figure 4). The plants treated with *E. cloacae* PM23 increased significantly (14–24%) in APX, (23–36%) in SOD, (26–36%) in POD, and (24–28%) in ascorbic acid activity under salinity stress (Figure 4). 

### 2.8. Effects of *E. cloacae* PM23 on Total Soluble Sugars, Proteins, Flavonoids and Phenolic Content of Leaves

**Figure 5:**
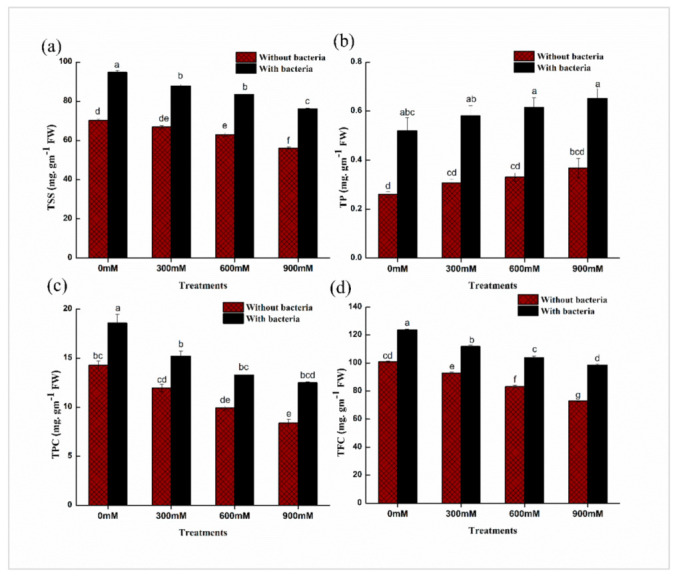
Effects of *E. cloacae* PM23 on (**a**) Total soluble sugars (**b**) Protein content (**c**) Phenolic content (**d**) Flavonoid content.

Total soluble sugar (TSS) content was decreased while total protein (TP) content was increased non significantly by progressive salinity stress in maize plants (Figure 5). After inoculating maize plants with *E. cloacae* PM23, a significant increase in TSS (24–26%) and protein content (45–50%) of leaves was observed in comparison with un-inoculated plants (Figure 5). While, in comparison with controls, flavonoids and phenolic contents were reduced under salinity stress of 300, 600, and 900 mM NaCl. With the inoculation of *E. cloacae* PM23, total flavonoid (20–28%) and phenolic content (28–36%) showed promising enhancement as compared to uninoculated plants (Figure 5).

### 2.9. Assessment of Potential Markers of Oxidative Stress and Compatible Solutes

Soil salinity proclaimed a promising influence on potential oxidative stress markers and compatible solutes (Table 4). Oxidative stress markers were increased under salinity stress (0, 300, 600 and 900 mM NaCl), while, *E. cloacae* PM23 inoculated treatments resulted in a significant decrease in electrolyte leakage (14–19%), H_2_O_2_ (18–25%), and malondialdehyde (MDA) content (50–56%) as compared to un-inoculated treatments (Table 4). Compared with the control plants, compatible solutes (Free amino acids, glycine betaine, and proline content) increased significantly with the application of *E. cloacae* PM23. The maize plants showed a significant increment in free amino acids (29–37%), glycine betaine (13–31%), and proline content (11–13%) as compared with non-inoculated ones (Table 5).

### 2.10. PCR Gene Amplification of Biotic and Abiotic Stress-Related Genes

**Figure 6:**
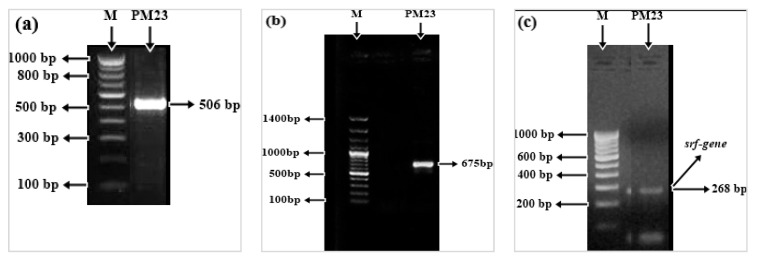
Amplification of biotic and abiotic stress-related genes: (**a**) *ItuC-gene* (**b**) *sfp-gene* (**c**) *srfAA-gene.* (M) represents marker.

Under salinity stress, genes responsible for salt stress tolerance in E. cloacae PM23 were examined. Polymerase chain reaction mediated amplification of Iturin C (*ItuC*) and bio-surfactant producing *sfp* and *srfAA* gene using the above-mentioned set of primers resulted in a sharp band of approximately 506, 675, and 268 base pairs (bp), respectively. These bands were observed on 2% agarose gel (Figure 6).

### 2.11. Gene Expression Analysis

**Figure 7:**
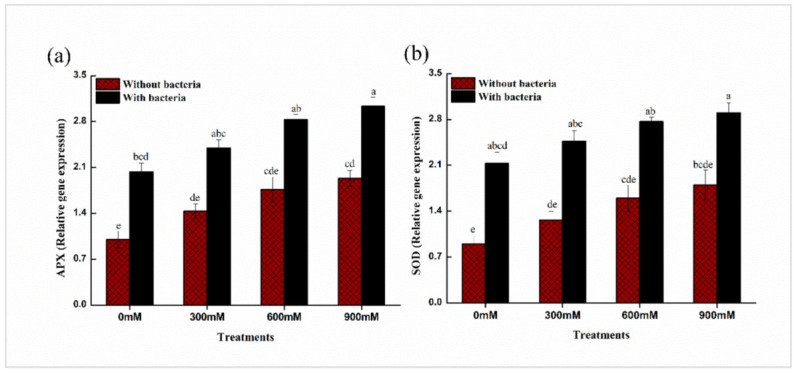
Expression levels of antioxidant genes of maize in the absence and presence of *E. cloacae* PM23 under salinity stress (**a**) Ascorbate peroxidase (APX) (**b**) Superoxide dismutase (SOD).

Compared to non-inoculated controls, *E. cloacae* PM23 inoculation upregulated two antioxidant genes (APX, SOD) (Figure 7). Furthermore, compared to non-inoculated salt-stressed plants, *E. cloacae* PM23 inoculated salinity-stressed maize plants showed significantly greater expression levels of antioxidant genes (Figure 7).

### 2.12. Principal Component and Pearson Correlation Analysis

**Figure 8:**
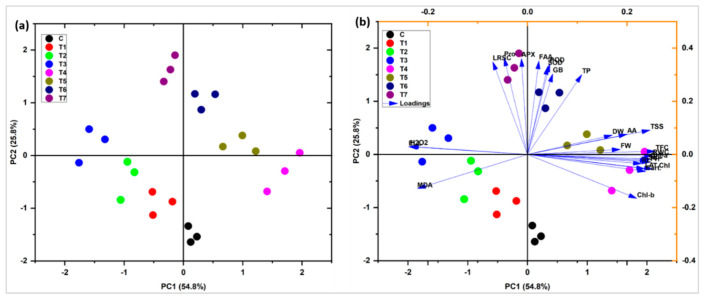
Categorization of *E. cloacae* PM23 based on its effects on maize growth-promoting characteristics under salinity stress (**a**) Cluster analysis (**b**) PCA Biplot analysis.

Principal component Biplot analysis showed a positive correlation between different variables under salinity stress with the application of *E. cloacae* PM23. The significantly correlated variables were placed very closely and in the same quadrate. Variable plot analysis showed 80.6% variations (PC_1_ = 54.8%; PC_2_ = 25.8%) (Figure 8). Shoot length (SL), fresh weight (FW), and root length (RL), carotenoids, total phenolic content (TPC), total flavonoid content (TFC), total soluble sugars (TSS), total protein (TP), relative water content (RWC), superoxide dismutase (SOD), peroxidases (POD), ascorbate peroxidase (APX), ascorbic acid (AA) and, osmoprotectants [free amino acids (FAA), glycine betaine (GB), proline] showed a positive correlation. On the contrary, the radical scavenging capacity (DPPH) of leaves and oxidative stress markers [electrolyte leakage (EL), hydrogen peroxide (H_2_O_2_), malondialdehyde (MDA)] were negatively correlated with all other variables.

**Figure 9:**
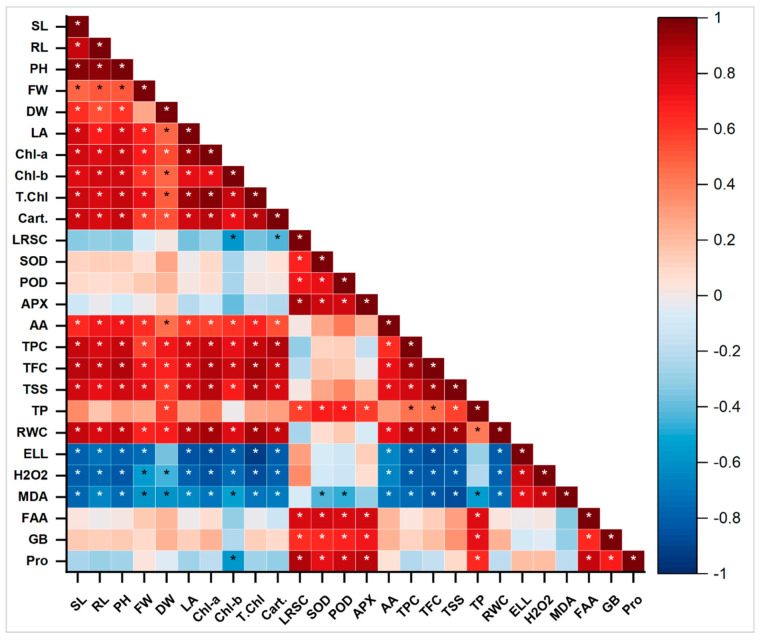
Pearson correlation between antioxidants and biochemical traits with plant biomass parameters under various salt stresses; Pro, (proline), SL (shoot length), RL (root length), PH (plant height), FW (fresh weight), DW (dry weight), LA (leaf area), Chl a (chlorophyll a), Chl b (chlorophyll b), T. chl (total chlorophyll), Caro (carotenoids), DPPH (radical scavenging capacity), SOD (superoxide dismutase), POD (peroxidases), APX (ascorbate peroxidase), AA (ascorbic acid), TPC (total phenolic content), TFC (total flavonoid content), TSS (total soluble sugars), TP (total protein), RWC (relative water content), EL (electrolyte leakage), H_2_O_2_ (hydrogen peroxide), MDA (malondialdehyde), FAA (free amino acids), GB (glycine betaine). The treatments exhibit (*) within rows that represent significance (*p* ≤ 0.05) level.

Pearson’s correlation of antioxidants and biochemical traits with plant biomass was analyzed (Figure 9). In maize plants, a highly positive correlation was observed between chlorophyll a, b, total chlorophyll, and carotenoids with SL, FW, and RL. Increasing these attributes directly correlated with the yield plant biomass and increased significantly (Figure 9). A strong positive correlation was found between total soluble sugar, relative water content, total phenolic content, chlorophyll a, b, total chlorophyll, and carotenoids with SL, FW, and RL. Similarly, higher POD, TP, GB, APX, SOD, FA, and proline were observed by increasing plant biomass (SL, RL, and FW), chlorophyll a, b, total chlorophyll, and carotenoids. The DPPH, EL, MDA, and H_2_O_2_ showed a strong negative correlation with all plant biomass attributes. On the contrary, the antioxidants and radical scavenging capacity, antioxidant enzymes, total phenolic content, total flavonoid content, total soluble sugars, total protein, electrolyte leakage, hydrogen peroxide, malondialdehyde, free amino acids, glycine betaine were observed in strong negative correlation. Decreasing the antioxidants also leads to a reduction in plant biomass under different treatments (Figure 9).

## 3. Discussion

Salinity stress affects plants’ physiological and biochemical mechanisms and pathways, causes an imbalance in nutrient uptake, alters growth-inducing regulators, and inhibits the synthesis of proteins and photosynthesis. These factors are responsible for reducing plant growth and yield [26]. The halo-tolerant microbial communities are of great agricultural importance to enhance crop productivity in both arid and semi-arid regions [27].

In the present investigation, the salinity tolerance potential of *E. cloacae* PM23 was evaluated. This bacterial strain indicated high tolerance towards salinity stress (3 M NaCl) (Figure 1). In our study, bacterial survivability was gradually declined with increasing salinity stress from 0 to 900 mM NaCl, which was in line with the previous study by Zhao et al. [28]. A substantial imbalance in an organism’s osmotic and water relation is caused by salinity stress, which declines the bacterial population [28]. The salt-tolerant (ST) *E. cloacae* PM23 produced floc yield and uptake of Na^+^ at a much higher rate under salinity stress (Figure 2). An increase in flocculation at various NaCl concentrations was confirmed with the previous report of Watanabe et al. [29], who observed that increasing NaCl concentration up to 6% increased the flocculation ability in marine photosynthetic bacterium *Rhodovulum* sp. by 80%. Salinity stress can be alleviated by using exopolysaccharides-producing plant growth promoting bacteria [20], as after binding with cation, EPS decreases bioavailable ions for plant uptake. Moreover, the results of our study depicted that biofilm formation of *E. cloacae* PM23 was significantly reduced under salinity stress (Figure 2). Reduced biofilm formation inhibited bacterial growth because of inhibitory osmotic stress [30,31].

Under salinity stress, *E. cloacae* PM23 significantly produced IAA, siderophore, ACC deaminase, and exopolysaccharides (Figure 3). In our study, *E. cloacae* PM23 produced IAA (41 µM/mL), promoted plant growth and yield by converting tryptophan into indole-3-acetic acid under stress (900 mM) (Figure 3). It was reported by Vimal et al. [32] that IAA when produced by the bacterial strains, directly facilitates the growth of the root via induction of cell elongation and/or response to cell division. Root development and structural changes to adapt under stress conditions are related to IAA production [33].

Siderophore production by *E. cloacae* PM23 increased significantly up to 109.44% under saline stress (900 mM) (Figure 3). Plants obtained iron from the soil and controlled phytopathogens with the help of siderophore-producing bacteria. Chlorophyll synthesis, electron transfer in photosynthetic and respiratory chain processes were regulated in plants by iron chelation [34]. DNA and RNA synthesis and their repairing improved by iron chelation [35].

Ethylene production regulates plant growth and development under salinity stress. Under salinity stress, *E. cloacae* PM23 secretes ACC deaminase (1.47 µM/mg Protein/h) at 900 mM salinity stress (Figure 3) which restricts ethylene biosynthesis. ACC deaminase enzyme converts ACC to ammonia and α-ketobutyrate. Inoculation with plant growth promoting rhizobacteria (PGPR) producing ACC overall improved the plant biomass by increasing the rate of photosynthesis while decreasing xylem equilibrium pressure [36].

In the current study, *E. cloacae* PM23 produced maximum EPS (4.5 mg/mL) at 900 mM NaCl, increasing significantly compared to non-saline conditions (Figure 3). Under salinity stress, bacterial EPS production is the most vital response to mitigate the stress [10]. Muhammad [37] also explained that EPS production was reduced under non-saline conditions and then increased with salinity. Some isolated halo-tolerant plant growth promoting bacteria increased EPS production and have a role in cell protection under salinity stress [38].

The decrease in plant growth and productivity was attributed to an increase in Na^+^ ion levels and oxidative stress, which harmed photosynthetic efficiency, ion imbalance, and membranes’ integrity [39]. Under salinity stress, *E. cloacae* PM23 inoculation overcomes the negative effects of salt stress on the growth and yield of maize plants (Table 2). Better performance of shoot, root, height, fresh, dry weight and leaf surface area was observed following inoculation with *E. cloacae* PM23 (Table 2). It might be related to higher ACC-deaminase activity of plant growth promoting rhizobacteria and enhanced root colonizing potential [40]. Rhizobacteria containing ACCD activity promoted the growth of inoculated plants under salinity stress by increasing electron transport and photosynthetic activity while lowering xylem balancing pressure and stomatal conductance [36].

Under salinity stress, chlorophyllase breakdowns the chlorophyll and other enzymes, which lowers photosynthetic pigment synthesis [41]. In terms of growth, biomass, photosynthetic pigments (chlorophyll a, b, and total chlorophyll), and carotenoid content, *E. cloacae* PM23 mitigated the detrimental effects of salinity stress in maize (Table 2 and Table 3). In addition to ACC-deaminase and EPS activity, *E. cloacae* enhanced growth hormone production (IAA), which may stimulate maize growth. High IAA synthesis by plant growth promoting rhizobacteria enhances the surface area and length of adventitious and lateral roots [42].

Recent findings demonstrated that maize leaf’s radical scavenging capacity (DPPH) was significantly enhanced by the inoculation of salt-tolerant plant growth promoting rhizobacteria *E. cloacae* PM23 under salinity stress (Table 3). Increased ROS scavenging ability by inoculating a plant with PGP microbes have been previously reported in canola [43], rice [18], chickpea [44], sunflower [45], and lettuce plants [46] under abiotic stress conditions. Osmotic stress is caused by salinity, one of the most prominent indicators of osmotic stress and decreased leaf relative water content [46]. Bacterial strain *E. cloacae* PM23 inoculated maize plants showed higher RWC than control (Table 3). These findings were parallel to previous Yang et al. [47], where Seeds of *Chenopodium quinoa* were pre-inoculated with halotolerant *Bacillus* sp. MN54 and *Enterobacter* sp. MN17 exhibited better salt tolerance and plant-water interactions.

Plants exhibit excellent oxidative stress defense mechanisms, including enzymatic antioxidants that inhibit ROS generation [48]. The antioxidant defense system (APX, POD, SOD, and ascorbic acid) can be improved to regulate reactive oxygen species (ROS), which induce oxidative stress under salinity stress. Our results demonstrated the promising enhancement in antioxidants with the inoculation of halo-tolerant *E. cloacae* PM23 under salinity stress (Figure 4). Under salt stress, several salt-tolerant plant growth promoting rhizobacteria including *Enterobacter cloacae*, *Pseudomonas pseudoalcaligenes*, and *Bacillus* sp. enhanced APX levels in Jatropha leaves, stimulated the roots, increased biomass, nutritional acquisition, and photosynthetic pigments in the plant’s vegetative regions [49].

POD is also important in eliminating H_2_O_2_ from seedling tissues and mitigating oxidative damage [50]. According to Elkelish et al. [51], SOD is the first line of defense against ROS, and it also inhibits the production of OH radicals, leading to lower lipid peroxidation in cell membranes [44]. Furthermore, ascorbic acid (AA) is an effective ROS scavenger due to its ability to produce enzymatic and non-enzymatic processes that regulate H_2_O_2_ and preserve cell membranes by scavenging free radicals [52].

Soluble solutes (Sugar and protein content) mitigate the lethal effects of salt stress and maintain ionic balance in cells [53]. The halo-tolerant *E. cloacae* PM23 exhibited a significant increase in flavonoid and polyphenol content under salinity stress (Figure 5). Plant growth promoting rhizobacteria inoculation greatly increased the activity of several ROS-scavenging enzymes in maize, basil, and rice [54]. Plant growth promoting rhizobacteria may elicit specific chemical changes in plants, such as changes in total protein, IAA concentration, total sugar, and ethylene content, improving abiotic stress tolerance, a process known as ‘induced systemic tolerance’ (IST) [55]. An salt-tolerant plant growth promoting rhizobacteria strain *Bacillus* sp. improved maize growth and development under drought and salinity [56].

Salinity stress significantly increased the free amino acids, glycine betaine, and proline content (Table 4). These osmolytes provide an osmoprotectant mechanism. In our investigation, *E. cloacae* PM23 inoculated plants exhibited a significant increment in maize plants under salinity stress (Table 4). ST- plant growth promoting rhizobacteria also uses this process to prevent osmotic stress, which is more prominent in soils affected by salinity [57]. Salt-tolerant bacteria may momentarily elevate their cytoplasmic K^+^ level in response to salt stress, however, osmolytes accumulation is a more frequent stress response to avoid water loss [58]. By lowering ROS detoxification caused by salt stress, proline content reduces ROS damage and improves plant tolerance [59]. *Azospirillum* spp. accumulates glycine betaine and proline, which helps the plant deal with osmotic stress [60].

In our results, antibiotic-resistant *Iturin C* (ItuC) and surfactant producing *sfp* and *srfAA* genes of *E. cloacae* PM23 were amplified (Figure 6). Bio-surfactants improve the interface between plant growth promoting rhizobacteria and the plant root, resulting in improved colonization and increased yield [61]. Furthermore, bio-surfactant may have assisted bacterial cells by improving their potential to form complexes with essential metal ions and micronutrients in soil by enhancing their nutrient chelating ability [62]. Furthermore, these bio-surfactants’ potent penetrating action, gelling, wetting, and amphiphilic properties make them an excellent dispersing agent [63], which could significantly benefit plant root colonization (by plant growth promoting rhizobacteria) and make phytohormones and siderophores available to the plant.

Furthermore, *E. cloacae* PM23 inoculation dramatically increased the expression of genes linked to salt tolerance and antioxidant enzyme-encoding genes (Figure 7). These findings are consistent with the findings of Elkelish et al. [51], who found that salt stress enhanced the expression of SOD and APX in chickpeas. Ji et al. [64] revealed that PGPR-inoculated rice seedlings had greater levels of antioxidant gene expression, which improved salt stress tolerance.

## 4. Materials and Methods

### 4.1. Procurement of Bacterial Strain

All bacterial strains (PM21, PM22, PM23, PM26, PM27, PM28, B29, B30, B31, B32, B33 and B38) were obtained from Plant-Microbe Interactions Lab, Quaid-i-Azam University, Islamabad, Pakistan. All these strains were evaluated against salt tolerance potential. From all bacterial strains, *Enterobacter cloacae* PM23 showed best salt tolerance potential under different concentrations (0, 1, 2, and 3 M) of NaCl [65]. *E. cloacae* PM23 tolerated up to 3 M NaCl and showed significant growth at all provided concentrations

### 4.2. Salinity Tolerance Characteristics of E. cloacae PM23

#### 4.2.1. Bacterial Survivability

The salt tolerance of halobiont bacterium *E. cloacae* PM23 was estimated based on the population density at different concentrations of salt (0, 300, 600, and 900 mM) in tryptic soy broth (TSB) medium. Sterilized flasks containing 100 mL TSB medium with different salt concentrations analyzed the bacteria. The TSB medium was inoculated with 10 μL of freshly prepared bacterial broth of *E. cloacae* PM23 and incubated at 26 ± 2 °C and 150 rpm in a shaking incubator. Ten ml of sterilized broth with both salt centration was incubated as an uninoculated control. After 24 h of incubation, the optical density of the culture was measured at λ = 600 nm using a spectrophotometer (Agilent 8453 UV–visible Spectroscopy System), and the growth was compared with un-inoculated control at a particular stress level [65].

#### 4.2.2. Bacterial Flocculation

To estimate bacterial flocculation, *E. cloacae* PM23 was grown in TSB medium with 0, 300, 600, and 900 mM NaCl for 72 h at 30 °C. The flocculation was collected using Whatman No. 1 filter paper and oven-dried at 60 °C. After 2 h, the dry weight of the floc yield was measured [66].

#### 4.2.3. Bacterial Sodium Absorption

A halotolerant *E. cloacae* PM23 was screened for sodium uptake capacity at different NaCl concentrations. *E. cloacae* PM23 was grown overnight at 30 °C in a TSB medium containing different NaCl concentrations (0, 300, 600, and 900 mM). The 24 h old bacterial cells were then centrifuged and harvested by centrifugation, and the bacterial pellet was washed with sterilized distilled water to remove the traces of medium. The washed pellet was before being digested overnight in 0.1 N HCl at room temperature. Centrifugation was performed to obtain the supernatant, and a flame photometer was used to assess bacterial sodium absorption [67].

#### 4.2.4. Biofilm Formation

The biofilm-forming capacity of *E. cloacae* PM23 was quantitatively analyzed by measuring the number of cells attached to a glass disk incorporated within the Petri dish containing the bacterial culture under different concentrations of NaCl (0, 300, 600, and 900 mM) [68], using the crystal violet staining method proposed by O’Toole and Kolter [69] with modifications. The glass was taken under aseptic conditions at each exposure time, washed with 1 mL of NaCl (0.9% *w*/*v*), and treated with 1 mL of crystal violet indicator (0.1% *w*/*v*) over 20 min. Then, the glasses were washed three times with NaCl (0.9% *w*/*v*). Biofilm formation was quantified by adding 1 mL of 95% ethanol to each crystal violet stained glass. Biofilm was quantified at 506/570 nm using a UV spectrophotometer (752 N UV-VIS, Beijing, China).

### 4.3. Quantitative Assay of Plant Growth-Promoting (PGP) Traits under Salinity Stress

#### 4.3.1. Estimation of Indole Acetic Acid (IAA)

Indole-3-acetic acid production was estimated by colorimetric assay [70]. A nutrient broth amended with 0.1% L-tryptophan and NaCl (0, 300, 600, and 900 mM) was inoculated with 1 mL of overnight grown bacterial culture. The culture broth was incubated in the shaker at 180 rpm for 48 h in the dark at 28 ± 2 °C. The bacterial culture was centrifuged at 10,000 rpm for 10 min at 4 °C. The supernatant (1 mL) was mixed with 4 mL Salkowski reagent and the appearance of a pink color indicated the production of IAA [71]. The absorbance of the final pink color solution was measured after 30 min at 535 nm in UV/Visible spectrophotometer and compared with the standard curve. The standard curve of IAA (Serva, Islandia, NY, USA) was made in the range of 10–100 µg/mL to estimate IAA concentration.

#### 4.3.2. Siderophore Production

The screening of *E. cloacae* PM23 for siderophore production was performed using CAS (Chrome azurol S) agar media [72]. For the preparation of CAS agar, 60.5 mg of CAS was dissolved in 50 mL of distilled water. Furthermore, 10 mL of Fe^3+^ solution (1 mM FeCl_3_·6H_2_O) and 40 mL of HDTMA (Hexadecyl trimethylammonium bromide) (72.9 mg in 40 mL dH_2_O) were dissolved in a previously made CAS solution. Agar (15 g) was added to the resultant dark blue solution and autoclaved. After the inoculation of the bacteria, plates were placed in an incubator for 7 d at 28 °C. The development of orange zones around the bacterial inoculation revealed positive results for siderophore production. To estimate siderophore production, the protocol of Mehmood et al. [73] was followed. At 630 nm, optical density was observed and siderophore was estimated as percent siderophore unit (PSU) using the following formula:

$$PSU=Ar-\frac{As}{Ar}\times 100$$

where, As is inoculated sample absorbance and Ar is a reference (un-inoculated broth + CAS reagent + salt conc.).

#### 4.3.3. Quantitative Estimation of ACC Deaminase

The ACC deaminase activity was quantified by following the method used by Zainab et al. [74]. Quantity of α-ketobutyrate produced by hydrolysis of ACC was used to estimate ACC deaminase. A standard curve of α-ketobutyrate was drawn ranging between 10–200 μmol and compared with absorbance taken at 540 nm of the sample, to determine μmol of α-ketobutyrate produced by this reaction.

#### 4.3.4. Exopolysaccharide Production (EPS)

The Exopolysaccharides quantification assay was performed following the method described in Zainab et al. [75]. Bacterial strain *E. cloacae* PM23 was grown in 50 mL ATCC no. 14-liquid medium: 0.2 g kH_2_PO_4_; 0.8 g K_2_HPO_4_; 0.2 g MgSO_4_·7H_2_O; 0.1 g CaSO_4_·2H_2_O; 2.0 mg FeCl_3_; Na 2MoO_4_·2H_2_O (trace); 0.5 g yeast extract, 20 g sucrose; with pH of 7.2 by using sucrose as a carbon source [76] supplemented with NaCl (0, 300, 600 and 900 mM). *E. cloacae* PM23 was also grown in 50 mL liquid medium ATCC no. 14 and incubated in a shaker for 3 days at 28 °C with 200 rpm rotation. At the end of incubation, bacterial cells were harvested in pellet form by adding 1 mM EDTA, homogenized by shaking, and centrifuged for 10 min at 9000 rpm. The supernatant containing EPS was separated and coupled with cold acetone with a proportion of 1:3. The mixture was again centrifuged at 15,000 rpm for 3 min. Deposition of EPS biomass was observed and washed with distilled water and dried until dry weights were fixed. The EPS was estimated as mg/mL of the dried weight.

### 4.4. Soil Collection, Analysis, and Seed Inoculation

The soil was collected from the Quaid-i-Azam University, Islamabad, Pakistan (33.7470° N, 73.1371° E). The soil was first air-dried in the laboratory, and then it was crushed, sieved, and sterilized to get rid of all microbes and fungal spores [65]. Soil physicochemical properties like electrical conductivity, pH, organic matter, soil texture, available phosphorus, and potassium were determined.

Certified maize seeds (SG-2002 variety) were collected from National Agricultural Research Center (NARC), Pakistan. Seeds were disinfected by serial washing with 70% ethyl alcohol for 5.0 min and 0.1% HgCl_2_ for 1.0 min. After disinfection, all seeds were rinsed three times with autoclaved distilled water. *E. cloacae* PM23 was cultured in 250 mL flasks containing LB broth. After 48 h, culture was taken and centrifuged for 10 min at 10,000 rpm to collect the pellet. The pellet was washed with 0.85% NaCl and resuspended in de-ionized water to maintain the absorbance at 0.5 and obtained a homogenous bacterial population [10^8^ colony-forming units (CFU) mL^−1^]. Seeds were dipped in bacterial solution for 2–4 h, while un-inoculated seeds were soaked in sterilized water taken as control [77].

### 4.5. Pot Experiment under Controlled Conditions

The Certified maize seeds (SG-2002 variety) were sown (6 surfaces sterilized seeds per pot) in plastic pots containing 200 g of sterilized soil Salt stress was applied to the plants after 5 days of germination once a day with 80, 120 and 180 mM NaCl increments to the plants until reaching final concentrations of 300, 600 and 900 mM NaCl in order to avoid osmotic shock stress.

Total of 8 treatments were organized in triplicate in a complete randomized design (CRD). Seeds were dipped in bacterial solution for 2–4 h, while un-inoculated seeds were soaked in sterilized water taken as control. The experimental design was as follows: (i) un-inoculated control plants I (ii) plants inoculated with *E. cloacae* PM23 (T1) (iii) 300 mM NaCl treated plants (T2) (iv) plants primed with 300 mM NaCl and *E. cloacae* PM23 (T3) (v) 600 mM NaCl-treated plants (T4); (vi) plants primed with 600 mM NaCl and *E. cloacae* PM23 (T5) (vii) 900 mM NaCl-treated plants (T6) (viii) plants spiked with 900 mM NaCl and *E. cloacae* PM23 (T7).

The pots were placId in a growth chamber (CU-36L6, Lowa, USA) for 21 days. Each treatment was received 20 mL of bacterial suspension after seed sowing with seven days of the interval (7, 14 days), while the control was treated with 20 mL sterilized broth medium.. Pots were rinsed with 50 mL of distilled water daily to maintain moisture for plant growth. Throughout the experiment, EC and the pH of the substrate in each pot were kept constant. The same quantity of water was sprayed regularly to maintain 60–70% water holding capacity, balancing NaCl levels in each pot. Photosynthetic photon flux density (PPFD) levels were maintained upto 350 µmol. Humidity was maintained up to 60–80 % in the growth chamber, the light duration for day and night was 12 h, and the temperature range was 32 °C and 20 °C for day and night, respectively.

*Zea mays* L. plants were harvested, and their roots were wiped under running tap water after a 21-day pot experiment to eliminate soil particles from the root surface. The soil was removed from the roots, bagged plants, and taken to the lab for further testing.

### 4.6. Estimation of Agro-Morphological Parameters of Zea Mays L.

Morphological parameters including plant height, length of shoot and root, fresh and dry biomass were analyzed for randomly selected three plants from each treatment and control after 21 d of cultivation. All the plants were dried in an 80 °C hot air oven for 24 h before being weighed. The total leaf area was calculated using the formula L × B × K, where L represents leaf length, B indicates leaf breadth, and K shows Kemp’s constant (For Monocot 0.9) [78].

### 4.7. Estimation of Photosynthetic Pigments of Plants

Photosynthetic pigments were extracted by homogenizing 0.1 g of fresh leaves with 6 mL of 80% ethanol. The extract was centrifuged, and the supernatant was taken in test tubes. A spectrophotometer (752 N UV-VIS, Beijing, China) was used to evaluate the optical density of chlorophyll a, b, and carotenoids at 663, 645, 510 and 480nm, respectively, [79] using the following formula:

Chlorophyll a =(12.7×A663)−(2.49×A645)Chlorophyll b =(12.9×A645)−(4.7×A663)Total chlorophyll =Chl a +Chl bCarotenoids =[(7.6×OD480) − 1.49 (OD510)] × [(Final volume of filtrate/1000) × 0.5)]

### 4.8. Relative Water Content (RWC) and Radical Scavenging Capacity of Leaves

The relative water content (RWC) of green leaves was calculated by determining the turgid weight of fresh leaf samples and drying them in a hot air oven until they reached a consistent weight [77]. A 0.5 g (FW) leaf was placed in a Petri dish filled with distilled water and left overnight in the dark. The turgid weight of the leaf was determined. After being heated at 72 °C overnight, the leaf’s dry weight (DW) was obtained.

RWC (%)= [FW−DW/TW−DW]×100

FW = Fresh Leaf weight; TW = Turgid leaf weight; DW = Dry leaf weight.

Radical scavenging activity or 2, 2-diphenyl-1-picrylhydrazyl (DPPH) of the extracts was evaluated according to the protocol of Asgari et al. [80]. Fresh leaves (100 mg) were crushed in 80% methanol, centrifuged at 10,000 rpm, and collected the supernatant. A suitable volume of supernatant (2 mL) was mixed with 180 µL of DPPH (Aldrich Chemistry, Burlington, US) solution (0.1 mM). After 30 min, the mixture was discolored, and optical density (OD) was measured with a spectrophotometer (752 N UV-VIS, Beijing, China) at 517 nm.

$$I\;\left(\%\right)=A_{c}-\frac{A_{s}}{A_{c}}\times 100$$

where A_c_ = Control; A_s_ = Sample’s absorbance.

### 4.9. Antioxidant Enzymatic Assays

Antioxidant activities of ascorbate peroxidase (APX), peroxidases (POD), and superoxide dismutase (SOD) were assessed in fresh leaves following the protocols of Hossain et al. [81] and Afridi et al. [65].

Fresh leaf samples (0.2 g) were crushed in a 2 mL extraction buffer (potassium phosphate, pH 7.5) and ascorbic acid (1 mM) to determine the APX level. The crushed materials were centrifuged for 20 min at 4 °C and 13,000 rpm. The OD was obtained at 290 nm to evaluate APX. A standard curve was used to measure the activity in units/mg proteins by estimating the decrement of ascorbate.

Using a precooled motor and pestle, freshly procured plant tissues (0.20 g) were crushed in 3 mL of 100 mM phosphate buffer (PB) for the POD assay. To separate the homogenate, the sample extract was centrifuged at 4 °C and 10,000 rpm for 15 min. To determine peroxidase, an OD at 470 nm was obtained. One unit of POD defined as the amount of enzyme that increases 0.100 of absorbance at 436 nm/min.

To estimate SOD, the plant material was crushed in 4 mL of solution (1 g PVP, 0.0278 g Na_2_EDTA) and centrifuged at 10,000 rpm. A reaction mixture (400 µL H_2_O + 350 µL phosphate buffer + 100 µL methionine + 50 µL NBT + 50 µL enzyme extract + 50 µL riboflavin) was prepared to measure the activity of the SOD enzyme. The mixture was then exposed to light for 15 min, with the decrease in absorbance measured at 560 nm. A blank was made by omitting the enzyme extract. The activities of SOD were then calculated and expressed in milligrams per milligram of total soluble protein.

The content of ascorbic acid (AA) in fresh leaves was determined using the protocol prescribed by El-Saadony et al. [82]. The result was derived using an ascorbic acid (Sigma-Aldrich, St. Louis, MO, USA) standard curve and expressed as mg/g FW.

### 4.10. Total Soluble Sugars (TSS) and Protein Content of Leaves

Total soluble sugars (TSS) were determined following the Grad method [83]. Optical density was measured at 625 nm with a spectrophotometer (752 N UV-VIS, Beijing, China), the TSS was estimated in µg/mL of fresh weight using the glucose standard curve.

The protein content of leaves was assessed in fresh leaves of maize using Bovine Serum Albumin (BSA) (Sigma-Aldrich, St. Louis, MO, USA) as a reference according to the described protocol of Mendez et al. [84]. The absorbance of all samples was measured at 750 nm, and protein content was estimated using BSA standard curve.

### 4.11. Total Flavonoids and Phenolic Content

The aluminum chloride colorimetric technique, revised from Woisky and Salatino’s method [85], was used to determine total flavonoid content (TFC). The calibration curve was generated using quercetin (Sigma, St. Louis, MO, USA). The quantitative results were computed in milligram of Quercetin equivalents per 100 gm fresh mass (mg QE/100). Each analysis was carried out three times [86].

The aluminum chloride colorimetric technique, revised from Woisky and Salatino’s method [85], was used to determine total flavonoid content (TFC). The calibration curve was generated using quercetin (Sigma, St. Louis, MO, USA). The quantitative results were computed in milligram of Quercetin equivalents per 100 gm fresh mass (mg QE/100). Each analysis was carried out three times [86].

A total of 5 mL of 70% methanol was added to the crushed leaves for homogenization. After 30 min incubation at 4 °C, the samples were centrifuged at 15,000 rpm for 10 min, and the supernatants were used for further analysis [87]. Total phenolic content was measured spectrophotometrically using a technique based on Folin-phenolic Ciocalteau’s reagent (Merck, Taufkirchen, Germany) [88]. Folin-reagent (0.5 mL) and 0.45 mL of 7.5% (*w*/*v*) saturated sodium carbonate solution were added to methanol extracted samples (20 µL). After a 2 h incubation period at 25 °C, samples’ absorbance at 765 nm was measured using a UV-VIS spectrophotometer (UV-9200, Beijing, China). Total phenolic compounds were computed and represented as mg gallic acid equivalent (mg GAE/100 g) sample using gallic acid (Sigma-Aldrich, St. Louis, USA) as a reference (100–800 mg/L). The absorbance of the reaction mixture was spectrophotometrically measured at 750 nm.

### 4.12. Valuation of Oxidative Stress Markers and Osmolytes

The electrolytes leakage from leaf discs was measured to calculate membrane stability index [89]. Leaf discs (0.10 g) of all treatments were placed in test tubes containing double distilled water. The EC of leaves were determined (C_1_) after 30 min, in a water bath at 40 °C. The same leaf sample was subsequently maintained in a water bath at 100 °C for 10 min, and the EC was measured again (C_2_).

Membrane Stability Index = [1 − C_1_/C_2_] × 100

Endogenous H_2_O_2_ content was determined according to method of Kapoor et al. [90]. Fresh weight (0.10 g) of leaf tissues was extracted with 3 mL of 0.1% trichloroacetic acid (TCA) in an ice bath and centrifuged at 12,000 rpm for 15 min to determine H_2_O_2_ concentration. A solution of 1 M potassium iodide (1.0 mL) and 10 mM potassium phosphate (0.50 mL) buffer (pH 7.00) were added to the supernatant. At 390 nm, the absorbance of the supernatant was measured. On a standardized curve, the content of H_2_O_2_ was expressed. On a standardized curve, the content of H_2_O_2_ was expressed.

Malondialdehyde (MDA) quantification was estimated followed by Tulkova [91]. In a cooled mortar and pestle containing 2 mL of 1% (*w*/*v*) trichloroacetic acid (TCA), a fresh leaf sample (0.2 g) was crushed. After centrifugation of 10 min at 15,000 rpm, 2 mL of the supernatant was removed and 4 mL of 0.5% thiobarbituric acid (TBA) was added to it. The mixture was heated to 95 °C and then allowed to cool. At 532 and 600 nm, the absorbance of all of the treated samples was determined. The quantity of TBA was calculated using the absorption co-efficient of 1.55 mmol/cm.

$$\mathrm{MDA}=\Delta \;(\mathrm{OD}532-\mathrm{OD}600)/1.56\;\times \;10^{5}$$

The free amino acid determination was performed using the ninhydrin technique described by Shafiq et al. [92]. Dried samples (200 mg) were homogenized in 5 mL of 80% alcohol and warmed for 15 min in a water bath. After that, the extract was centrifuged for 20 min at 2000 rpm. In a water bath, 0.20 mL sample of the reaction mixture was heated with 3.80 mL of ninhydrin reagent. The reaction mixture was cooled until it became purple blue. At 570 nm, absorbance was measured. The standard curve was constructed using leucine amino acid, and findings were reported in mg of amino acid per gram of dry tissue.

The glycine betaine (GB) content was determined following previously published protocol [93]. The dried sample (500 mg) was homogenized in an extract prepared by mixing 5 mL of distilled water and 0.05% toluene for 24 h. The reaction mixture was filtered using 0.2 mm micropore filters or centrifuged at 6000 rpm for 5 min. A 0.5 mL sample of this extract was thoroughly mixed with 1 mL of HCl (2 N) and 0.1 mL of KI. The mixture was kept on ice for 2 h and vigorously shaken. This extract was gently mixed with 2 mL of ice-cold water and 10 mL of 1, 2-Dichloroethane or Dichloromethane. Two layers were formed, and the upper aqueous layer was removed. The optical density of the bottom pink-colored layer was recorded at 365 nm. The glycine betaine content in µg/gm dry weight was estimated by plotting a standard curve using betaine hydrochloride.

For the measurement of proline content in shoots, the method of Parveen and Siddiqui [94] was used. Fresh shoot material (0.2 g) was crushed in 3 mL of 3% sulphosalicylic acid, stored at 5 °C overnight. The obtained suspension was centrifuged for 5 min at 3000 rpm. Supernatant (2 mL) was blended with acidic ninhydrin reagent after centrifugation. This reagent was made by dissolving 1.25 g ninhydrin in 20 mL phosphoric acid (6 M) and 30 mL glacial acetic acid (1 M H_3_PO_4_ = 3 N H_3_PO_4_) with constant stirring. The reagent was kept stable for 24 h. The tubes carrying the contents were heated for 1 h in a water bath at 100 °C. After cooling, mixture was extracted with 4 mL toluene in a separate funnel. At 520 nm, optical density was determined using toluene as a blank.

Proline µg/g = K × DF × Absorbance/FW

K = 17.52; Dilution factor = 2; Fresh weight = 0.5 g.

### 4.13. Amplification of Iturin C (ItuC) and Bio-Surfactant Producing Genes

The primer *Iturin C* (ItuC) was used to detect antibiotic biosynthesis gene [95]. PCR reaction was done in a 25-μL reaction mixture. Thermal cycling conditions were initial activation at 95 °C for 15 min, 35 cycles of 95 °C for 1 min, 58 °C for 1 min, and 72 °C for 1.5 min, and at 72 °C for 7 min. The following forward primer: ITUC-F1 5′-CCCCCTCGG TCAAGTGAATA-3′; Reverse primer: ITUC-R1 5′-TTGGTTAAG CCCTGATGCTC-3′ were used.

PCR was used to amplify the *sfp* gene from genomic DNA using two oligonucleotide primers [96]; Forward Primer: sfp F: 5′ ATGAAGATTTACGGAATTTA-3′ and Reverse Primer: sfp R: 5′-TTATAAAAGCTCTTCGTACG-3′. The thermal cycler was set for an initial denaturation cycle of 1 min at 94 °C, followed by 25 cycles of 1 min denaturation at 94 °C, 30 sec annealing at 46 °C, 1 min extension at 72 °C, and a 10 min final extension at 72 °C [96].

Similarly, PCR was used to amplify the *srfAA* gene (268 bp) that encodes surfactant production by using two primers Forward primer; F-5′-TCGGGACAGGAAGACATCAT-3′; Reverse primer: R-5′-CCACTCAAACGGATAATCCTGA-3′ [97]. The primer’s annealing temperature was set to 58–60 °C. During electrophoresis, a 2% agarose gel was used to examine the PCR product. Sharp bands of the aforementioned genes were detected in the Gel Doc system (Universal Hood II, California, CA, US).

### 4.14. Gene Expression Analysis of Antioxidant (APX and SOD) Genes in E. cloacae PM23

The expression level of antioxidant genes (APX and SOD) was quantified by using quantitative real-time PCR (qRT-PCR) in the presence and absence of *E. cloacae* PM23 under salinity stress (0, 300, 600, and 900 mM NaCl). Qiagen RNeasy Plant Mini kit was used to isolate total RNA from maize plant and contaminated DNA was removed. Qiagen Reverse Transcription kit was used to synthesize cDNA. PCR amplification conditions were set up as described by El-Esawi et al. [98]. Specific primers previously designed for the 2 antioxidant genes were used for amplification [44]. The housekeeping gene Actin was utilized, and the genes expression level was determined following the 2^−ΔΔCt^ method.

### 4.15. Statistical Analysis

For each treatment, all data were obtained, and mean values and standard errors were computed. Data were analyzed using analysis of variance (ANOVA) and pairwise comparison among treatment means was made by LSD test at *p* = 0.05 using Statistix 8.1. Principal Component Analysis (PCA) and Pearson correlation analysis was applied using XLStat and R-software.

## 5. Conclusions

The present study demonstrated that the inoculation of halo-tolerant bacterium *E. cloacae* PM23 increased the resistance of maize plants under elevated salt concentrations. The halo-tolerant bacterium exhibits PGP properties, including biofilm formation, IAA, siderophore ACC deaminase, and EPS, and applied as a liquid formulation, enhancing maize growth and biomass compared to control under salinity stress. The strain *E. cloacae* PM23 also produced proline and antioxidant enzymes, which resulted in a decrease in oxidative stress on maize plants measured in terms of H_2_O_2_ and MDA content. The strain *E. cloacae* PM23 showed a promising role in mitigating salinity stress by modulating the antioxidant defensive system, reducing oxidative stress markers, and accumulating soluble and compatible solutes in maize plants. Moreover, molecular profiling and expression of stress-related genes of *E. cloacae* PM23 supported its role in promoting plant growth under salinity stress and multi-stress tolerance. Plant growth promoting rhizobacteria isolated from the rhizosphere of various plants revealed several growth-promoting traits and induced resistance in plants under saline stressed environments. As a result, finding novel and efficient bio-inoculants to improve crop yields has become critical. Evaluation from the current study suggests that halo-tolerant *E. cloacae* PM23 can potentially be utilized as a promising alternative and environmentally friendly approach to facilitate maize’s growth and salt tolerance in salinity stress. This isolated, tested *E. cloacae* PM23 presented the striking potential for use as a PGPR in controlled conditions under salinity stress. Moreover, field experiments are required to evaluate its full potential to mitigate salinity stress.

## Figures and Tables

**Table 1:** Physicochemical properties of soil.

Soil Parameters	Soil 1 (Pre-Sowing)	Soil 2 (Post-Harvesting)
Soil texture	Loamy	Loamy
pH	7.94	7.87
Electrical conductivity (dS/m)	1.53	4.49
Organic matter (%)	3.49	1.88
Available Phosphorus (mg/kg)	45.62	33.85
Available Potassium (mg/kg)	601	124
Saturation (%)	44	43

**Table 2:** Maize growth, biomass and leaf surface area in presence and absence of *Enterobacter cloacae* PM23 under salinity stress.

NaCl (mM)	*E. cloacae * PM23	SL (cm)	RL (cm)	PH (cm)	FW (g)	DW (g)	LA (cm^2^)
0 mM	−PM23	30.4 ± 1.22 bc	13.55 ± 0.5 b	43.95 ± 1.01 bc	1.30 ± 0.19 abc	0.5 ± 0.10 ab	15.01 ± 0.58 bc
	+PM23	38.5 ± 0.88 a	18.9 ± 1.10 a	57.4 ± 1.71 a	2.15 ± 0.11 a	0.93 ± 0.08 a	20.75 ± 1.24 a
300 mM	−PM23	26 ± 1.06 cd	10.83 ± 0.42 bc	36.83 ± 1.47 cd	0.93 ± 0.16 c	0.42 ± 0.06 ab	13.22 ± 0.42 c
	+PM23	34.2 ± 1.13 ab	15 ± 1.21 ab	49.2 ± 2.35 ab	1.83 ± 0.15 ab	0.86 ± 0.13 a	18.51 ± 0.88 ab
600 mM	−PM23	22 ± 0.79 de	7.87 ± 0.41 cd	29.87 ± 1.09 de	0.79 ± 0.14 c	0.37 ± 0.07 ab	11.46 ± 0.27 cd
	+PM23	30.45 ± 1.09 bc	13.25 ± 0.80 b	43.7 ± 1.05 bc	1.44 ± 0.12 abc	0.75 ± 0.11 ab	14.54 ± 0.56 bc
900 mM	−PM23	16.33 ± 1.19 e	6 ± 0.43 d	22.33 ± 1.63 e	0.62 ± 0.06 c	0.26 ± 0.03 b	8.36 ± 0.31 d
	+PM23	25.38 ± 1.17 cd	10.9 ± 0.22 bc	36.28 ± 1.34 cd	1.26 ± 0.06 bc	0.62 ± 0.06 ab	11.34 ± 0.17 cd

Growth was measured at 21 days after seed owing under different salt concentration regimes. SL–shoot length, RL–root length, PH–Plant height, FW–fresh weight, DW–dry weight. The treatments exhibit dissimilar letters within rows that represent significance (*p* ≤ 0.05) level.

**Table 3:** Influences of *E. cloacae* PM23 on chlorophyll a, b, total chlorophyll, carotenoids and relative water content in leaves under salinity stress.

NaCl (mM)	*E. cloacae * PM23	Chl a (mg/g FW)	Chl b (mg/g FW)	Total Chl (mg/g FW)	Carotenoids (mg/g FW)	RWC (%)
0 mM	−PM23	17.1 ± 0.90 cd	5.44 ± 0.10 ab	22.54 ± 0.79 b	6.01 ± 0.20 bc	55.43 ± 0.74 cd
	+PM23	25.16 ± 0.80 a	6.1 ± 0.16 a	31.26 ± 0.67 a	8.58 ± 0.15 a	72.34 ± 0.95 a
300 mM	−PM23	14.05 ± 0.32 cde	4.50 ± 0.12 c	18.55 ± 0.19 bcd	5.43 ± 0.12 bcd	50.73 ± 1.15 de
	+PM23	21.96 ± 0.83 ab	5.31 ± 0.1 b	25.2 ± 0.58 b	6.5 ± 0.17 b	66.27 ± 1.07 b
600 mM	−PM23	12.36 ± 0.55 ef	3.51 ± 0.11 de	15.87 ± 0.52 d	4.77 ± 0.13 de	47.21 ± 0.81 ef
	+PM23	17.84 ± 0.48 bc	4.29 ± 0.11 cd	22.14 ± 0.43 bc	5.57 ± 0.14 bcd	59.41 ± 1.09 c
900 mM	−PM23	9.33 ± 0.39 f	2.39 ± 0.13 e	11.72 ± 0.49 e	3.91 ± 0.21 e	44.22 ± 0.79 f
	+PM23	13.48 ± 0.57 def	3.46 ± 0.08 f	16.95 ± 0.52 cd	4.96 ± 0.21 cde	50.94 ± 0.67 de

The chlorophyll contents in leaves and relative water contents were measured after 21 days of seed sowing. Chl a–chlorophyll a, Chl b–chlorophyll b, Total Chl–total chlorophyll and carotenoids. The treatments exhibit dissimilar letters within rows that represent significance (*p* ≤ 0.05) level.

**Table 4:** Level of oxidative stress markers in presence and absence of *E. cloacae* PM23 under salinity stress.

NaCl (mM)	*E. cloacae* PM23	DPPH (IC_50_) %	EL (%)	H_2_O_2_ (µmol/g FW)	MDA (nmol/g FW)
0 mM	−PM23	30.80 ± 0.64 e	45.4 ± 1.09 cd	28.50 ± 0.60 cd	9.73 ± 0.30 bc
	+PM23	35.81 ± 0.42 d	39.1 ± 0.81 e	21.36 ± 0.66 e	4.32 ± 0.22 d
300 mM	−PM23	35.15 ± 1.10 d	51.33 ± 0.66 bc	31.24 ± 0.92 bc	10.54 ± 0.34 b
	+PM23	40.42 ± 0.18 c	43.53 ± 1.17 de	25.53 ± 0.22 de	5.28 ± 0.55 d
600 mM	−PM23	42.97 ± 0.80 c	56.6 ± 1.34 b	35.72 ± 1.09 ab	12.02 ± 0.47 ab
	+PM23	58.27 ± 0.19 a	45.8 ± 0.81 cd	29.38 ± 1.06 cd	5.72 ± 0.49 d
900 mM	−PM23	48.74 ± 0.87 b	63.43 ± 0.41 a	38.63 ± 0.89 a	14.24 ± 0.96 a
	+PM23	60.31 ± 0.17 a	51.96 ± 0.82 b	31.08 ± 1.05 bcd	6.49 ± 0.11 cd

The effect of NaCl treatments under different salt concentration conditions. DPPH–Radical scavenging activity of leaves, EL–Electrolyte leakage, H_2_O_2_–Hydrogen peroxide, MDA–Malondialdehyde. The treatments exhibit dissimilar letters within rows that represent significance (*p* ≤ 0.05) level.

**Table 5:** Level of osmolytes in presence and absence of *E. cloacae* PM23 under salinity stress.

NaCl (mM)	*E. cloacae * PM23	FAA (mg/g DW)	GB (µg/g DW)	Proline (µmol/g FW)
0 mM	−PM23	13.39 ± 1.25 d	8.01 ± 0.43 d	60.48 ± 1.06 f
	+PM23	18.26 ± 1.35 cd	11.63 ± 0.16 abc	68.96 ± 0.80 de
300 mM	−PM23	15.97 ± 0.93 d	8.82 ± 0.40 cd	65.88 ± 1.06 ef
	+PM23	22.67 ± 0.66 bc	12.09 ± 0.39 ab	73.8 ± 1.48 cd
600 mM	−PM23	17.25 ± 0.79 cd	9.43 ± 0.53 bcd	70.39 ± 1.05 de
	+PM23	26.78 ± 0.94 ab	12.64 ± 0.52 a	80.47 ± 0.40 ab
900 mM	−PM23	19.42 ± 0.78 cd	11.78 ± 0.52 abc	78.53 ± 1.05 bc
	+PM23	30.86 ± 0.79 a	13.56 ± 0.66 a	86.55 ± 0.69 a

The effect of NaCl treatments under different salt concentration conditions. FAA–Amino Acid, GB–Glycine betaines. The treatments exhibit dissimilar letters within rows that represent significance (*p* ≤ 0.05) level.

## Data Availability

The paper reflects the authors’ own research and analysis in a truthful and complete manner. The paper is not currently being considered for publication elsewhere. All authors have been personally and actively involved in substantial work leading to the paper, and will take public responsibility for its content.
